# Supplementary material for: Post-remission outcomes in AML patients with high hyperleukocytosis and inaugural life-threatening complications
Source: PLoS One. 2022 Jul 7;17(7):e0270744. doi: 10.1371/journal.pone.0270744 (PMC9262217; doi:10.1371/journal.pone.0270744)
Supplement: Attachment — Submitted filename: point-by-point responses.docx [file pone.0270744.s002.docx]

Response to Edits requested on your revised submission

1. Your ethics statement should only appear in the Methods section of your manuscript. If your ethics statement is written in any section besides the Methods, please move it to the Methods section and delete it from any other section. Please ensure that your ethics statement is included in your manuscript, as the ethics statement entered into the online submission form will not be published alongside your manuscript.

It has been changed according to your comments

2. Please ensure that you refer to Table 5 in your text as, if accepted, production will need this reference to link the reader to the Table.

   It has been changed according to your comments
3. In line with our goal of ensuring long-term data availability to all interested researchers, PLOS’ Data Policy states that authors cannot be the sole named individuals responsible for ensuring data access (<http://journals.plos.org/plosone/s/data-availability#loc-acceptable-data-sharing-methods>).

We provided the raw data of the trial as requested

We thank the editor and 3 reviewers for their constructive comments.

- Journal Requirements:

1. Please ensure that your manuscript meets PLOS ONE's style requirements, including those for file naming. The PLOS ONE style templates can be found at

<https://journals.plos.org/plosone/s/file?id=wjVg/PLOSOne_formatting_sample_main_body.pdf>  and

<https://journals.plos.org/plosone/s/file?id=ba62/PLOSOne_formatting_sample_title_authors_affiliations.pdf>

We checked and made sure that our manuscript meets PLOS ONE’s style requirements

2.  Please provide additional details regarding participant consent. In the ethics statement in the Methods and online submission information, please ensure that you have specified what type you obtained (for instance, written or verbal, and if verbal, how it was documented and witnessed). If your study included minors, state whether you obtained consent from parents or guardians. If the need for consent was waived by the ethics committee, please include this information.

We clarified this point and add a sentence to the Methods section “According to the French law on non-interventional study on health data, patients provided a written non-opposition statement for anonymous data collection. This study was approved by the institutional review board of the French society of intensive care medicine (CE SRLF 21-88).”

3. Please delete the separate files, leaving only the tables in the manuscript file

The changes were made according to the editor's comments

4. Please include a copy of Table 5 which you refer to in your text on page 28.

The changes were made according to the editor's comments

5. We note that you have stated that you will provide repository information for your data at acceptance. Should your manuscript be accepted for publication, we will hold it until you provide the relevant accession numbers or DOIs necessary to access your data. If you wish to make changes to your Data Availability statement, please describe these changes in your cover letter and we will update your Data Availability statement to reflect the information you provide.

We changed the data availability statements and specified that our data will be only available upon direct request to the corresponding author

**Comments to the Author**

1. Is the manuscript technically sound, and do the data support the conclusions?

The manuscript must describe a technically sound piece of scientific research with data that supports the conclusions. Experiments must have been conducted rigorously, with appropriate controls, replication, and sample sizes. The conclusions must be drawn appropriately based on the data presented.

Reviewer #1: Yes

Reviewer #2: Yes

2. Has the statistical analysis been performed appropriately and rigorously?

Reviewer #1: Yes

Reviewer #2: Yes

3. Have the authors made all data underlying the findings in their manuscript fully available?

The [PLOS Data policy](http://www.plosone.org/static/policies.action#sharing) requires authors to make all data underlying the findings described in their manuscript fully available without restriction, with rare exception (please refer to the Data Availability Statement in the manuscript PDF file). The data should be provided as part of the manuscript or its supporting information, or deposited to a public repository. For example, in addition to summary statistics, the data points behind means, medians and variance measures should be available. If there are restrictions on publicly sharing data—e.g. participant privacy or use of data from a third party—those must be specified.

Reviewer #1: Yes

Reviewer #2: Yes

4. Is the manuscript presented in an intelligible fashion and written in standard English?

PLOS ONE does not copyedit accepted manuscripts, so the language in submitted articles must be clear, correct, and unambiguous. Any typographical or grammatical errors should be corrected at revision, so please note any specific errors here.

We would like to thank the reviewers once again for their time in reading and comments on our manuscript

Reviewer #1: No. Corrections have been made accordingly in the manuscript

Reviewer #2: Yes

5. Review Comments to the Author

Please use the space provided to explain your answers to the questions above. You may also include additional comments for the author, including concerns about dual publication, research ethics, or publication ethics. (Please upload your review as an attachment if it exceeds 20,000 characters)

Reviewer #1: In the manuscript "Post-remission outcomes in AML patients with high hyperleukocytosis and inaugural life-threatening complications“ the authors investigated a large retrospective cohort of patients with AML presenting initially with hyperleucocytosis. The study is clinically relevant and the statics are performed correctly. However, there is still a need for thorough language editing.

We thank the reviewer 1 for his comments, language editing has been made according to reviewer 1 comment

Reviewer #2: The manuscript is clear, concise and well written. I recommend this manuscript be accepted.
Minor comments
-In the abstract part of the paper, please include about the statistical analysis

We thank the reviewer 2 for his comments.

We included a sentence about the statistical analysis in the abstract.

-In the method part of the document, you have to provide the data collection procedures. Please also define your study variables (dependent vs independent variable) clearly.

We clarified on how and which the data were collected in the methods part of the manuscript : “Datasets were anonymously extracted from medical charts and laboratory records. We collected clinical data at diagnosis and at the last visit for living patients, biological data on haematology, biochemistry and haemostasis and finally biological data on the characteristics of the leukaemia. For each patient, we also collected the treatments implemented as well as the different life sustaining therapies employed.”

-Some references are too old (before 2000) and need to be updated

Modifications has been made, according to the reviewer comment

6. PLOS authors have the option to publish the peer review history of their article ([what does this mean?](https://journals.plos.org/plosone/s/editorial-and-peer-review-process#loc-peer-review-history)). If published, this will include your full peer review and any attached files.


If you choose “no”, your identity will remain anonymous but your review may still be made public.


**Do you want your identity to be public for this peer review?** For information about this choice, including consent withdrawal, please see our [Privacy Policy](https://www.plos.org/privacy-policy).

Reviewer #1: No

Reviewer #2: No
